# Clinical Features and Laboratory Examination to Identify Severe Patients with COVID-19: A Systematic Review and Meta-Analysis

**DOI:** 10.1155/2021/6671291

**Published:** 2021-11-15

**Authors:** Yan Meng, Jinpeng Wang, Kaicheng Wen, Wacili Da, Keda Yang, Siming Zhou, Zhengbo Tao, Hang Liu, Lin Tao

**Affiliations:** ^1^The First Hospital of China Medical University, Shenyang, 110001 Liaoning, China; ^2^Ragon Institute of MGH, MIT and Harvard, USA; ^3^Institute of Health Sciences of China Medical University, Shenyang, 110001 Liaoning, China

## Abstract

**Background:**

With the COVID-19 epidemic breakout in China, up to 25% of diagnosed cases are considered to be severe. To effectively predict the progression of COVID-19 via patients' clinical features at an early stage, the prevalence of these clinical factors and their relationships with severe illness were assessed.

**Methods:**

In this study, electronic databases (PubMed, Embase, Web of Science, and Chinese database) were searched to obtain relevant studies, including information on severe patients. Publication bias analysis, sensitivity analysis, prevalence, sensitivity, specificity, likelihood ratio, diagnosis odds ratio calculation, and visualization graphics were achieved through software Review Manager 5.3, Stata 15, Meta-DiSc 1.4, and R.

**Results:**

Data of 3.547 patients from 24 studies were included in this study. The results revealed that patients with chronic respiratory system diseases (pooled positive likelihood 6.07, 95% CI: 3.12-11.82), chronic renal disease (4.79, 2.04-11.25), cardiovascular disease (3.45, 2.19-5.44), and symptoms of the onset of chest tightness (3.8, 1.44-10.05), shortness of breath (3.18, 2.24-4.51), and diarrhea (2.04, 1.38-3.04) exhibited increased probability of progressing to severe illness. C-reactive protein, ratio of neutrophils to lymphocytes, and erythrocyte sedimentation rate increased a lot in severe patients compared to nonsevere. Yet, it was found that clinical features including fever, cough, and headache, as well as some comorbidities, have little warning value.

**Conclusions:**

The clinical features and laboratory examination could be used to estimate the process of infection in COVID-19 patients. The findings contribute to the more efficient prediction of serious illness for patients with COVID-19 to reduce mortality.

## 1. Introduction

Coronavirus disease (COVID-19), which is caused by the 2019 novel coronavirus (SARS-CoV-2), broke out first in Wuhan, Hubei Province, China, in December 2020. Since then, the virus has quickly spread throughout all over the world. Human-to-human transmission of COVID-19 has been confirmed, and the virus can be transmitted through droplets and contact; it may also be spread via fecal-oral, blood, and aerosol transmission. COVID-19 may be asymptomatic, but the disease may progress rapidly; the proportion of severe patients is as high as 25.5% among confirmed patients and 76% among suspected patients [1]. COVID-19 has spread throughout the world, and many countries with poor medical conditions cannot make full use of the existing medical conditions to effectively fight the epidemic. Therefore, accurately predicting the occurrence and progress of COVID-19 and making treatment preparation in advance are of great significance for disease treatment. Patients especially vulnerable to severe illness are with preexisting comorbidities ones such as COPD, diabetes, cardiovascular diseases, and chronic renal disease [2]. However, no clinical characteristic differences or correlations have been found between severe and nonsevere cases. The main purpose of this study is to present systematic reviews and detailed estimates of the major symptoms of COVID-19, determine the prevalence of serious comorbidities, and assess patient progression via comorbidities, major symptoms, and laboratory examination to help severe patients and effectively reduce the mortality of COVID-19 under the condition of limited medical resources.

## 2. Methods

### 2.1. Search Strategy and Selection Criteria

**Figure 1:**
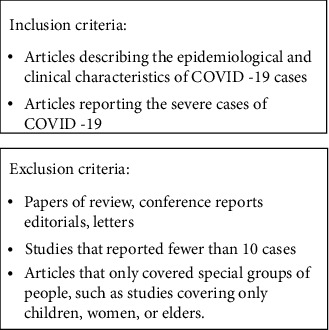
Inclusion and exclusion criteria.

The PubMed, Embase, Web of Science, and Chinese databases were searched using the term “((COVID-19) OR (2019 nCov) OR (2019 coronavirus)) AND ((ICU) OR (severe)) AND ((clinical characteristics) OR (clinical feature))” on April 7, 2020, without limiting the language of the articles, including those describing the epidemiological and clinical characteristics of COVID-19 cases and excluding articles of review, conference reports, editorials, letters, etc. In addition, studies that included data on fewer than 10 cases were excluded (Figure 1). References listed in the retrieved articles were also browsed to prevent the overlooking of relevant research. According to the inclusion and exclusion criteria, two researchers independently conducted literature screening. Any disagreements between the two researchers were resolved through discussions with a third independent researcher. The retrieved articles underwent two rounds of screening, the first of which was based on the titles and abstracts, and the second of which was based on the full texts of the articles.

### 2.2. Quality Assessment

We use the QUADAS-2 tool in Review Manager 5.2 to evaluate the quality of literature. The QUADAS-2 tool evaluates the quality of the included literature from four aspects: patient selection, index test, reference standard, and flow and timing.

### 2.3. Data Extraction and Analysis

The data of each included study was extracted by one researcher, then verified and proofread by a second researcher. The two researchers corrected all errors through discussion and proofreading. General information about the patients' genders and clinical characteristics such as symptoms, signs, chronic comorbidities, laboratory test results, and whether they were severe cases was extracted.

To assess the prevalence of clinical features in COVID-19 cases, a meta-analysis of single rate of each clinical feature was performed. It was predicted that comorbidities would differ between different populations and that the studies were heterogeneous. Therefore, this meta-analysis was performed using a random effects model. Cochran's *Q* test was then performed with Stata 15, and the value of the degree of heterogeneity *I*^2^ was calculated to verify the correctness of the random effects model. When *p* ≤ 0.1 or *I*^2^ ≥ 50%, it indicated that it was correct to adopt the random effects model; otherwise, the fixed effect model was used. Finally, a forest map of comorbidities was created to show the prevalence of each clinical feature in patients.

To evaluate the diagnostic values of different clinical features of patients for future critical care, 2 × 2 tables were constructed for each clinical feature and each situation of being severe illness based on the extracted information. When an item in the table was empty, 0.5 was added to all cells. The sensitivity, specificity, positive likelihood ratio, negative likelihood ratio, and diagnostic ratio of each clinical feature, which can indicate the effect of the presence or absence of clinical features on the possibility of requiring critical care, were then calculated with a 95% confidence interval. A higher positive likelihood ratio indicates that the presence of clinical features can increase the possibility of the patient becoming severe in the future; conversely, a lower negative likelihood ratio indicates that the absence of clinical features can reduce the possibility of the patient becoming severe.

Publication bias analysis and sensitivity analysis were performed for each indicator to be evaluated through Stata 15. All the above calculations and drawings are realized by software Review Manager 5.3, Stata 15, Meta-DiSc 1.4, and R.

## 3. Results

**Figure 2:**
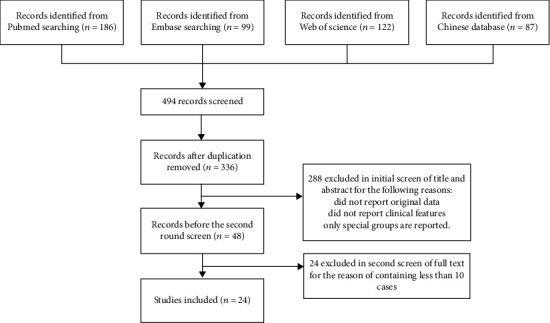
Work flow for the article selection.

Figure 2 presents the work flow for the selection of the included 24 studies with 3,547 patients in total [3–26]. All studies were published in 2020. The basic information of the included studies is shown in Table 1. The number of cases in the selected studies varied by ~64.6-fold and ranged from 17 to 1,099 cases. The median prevalence of severe patients was found to be 30% (95% CI: 25-35%). The proportion of male patients was 54% (95% CI: 50-57%), with the male-to-female sex ratio 1.8.

**Figure 3:**
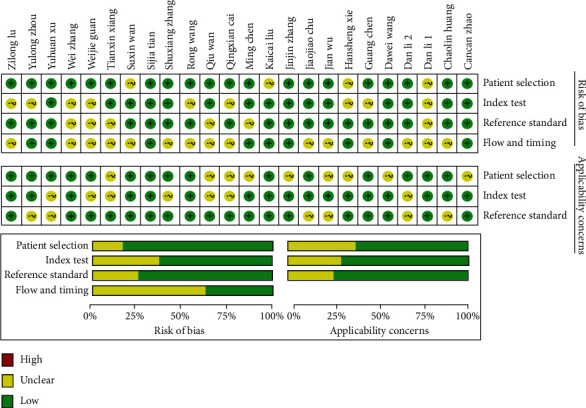
Quality assessment of the included articles.

Quality assessment of the articles is shown in Figure 3. Most studies have lower bias and higher quality. A small number of articles may have problems with the time flow of the occurrence of symptoms and confirmation of severe illness, affecting the quality of the study.

**Figure 4:**
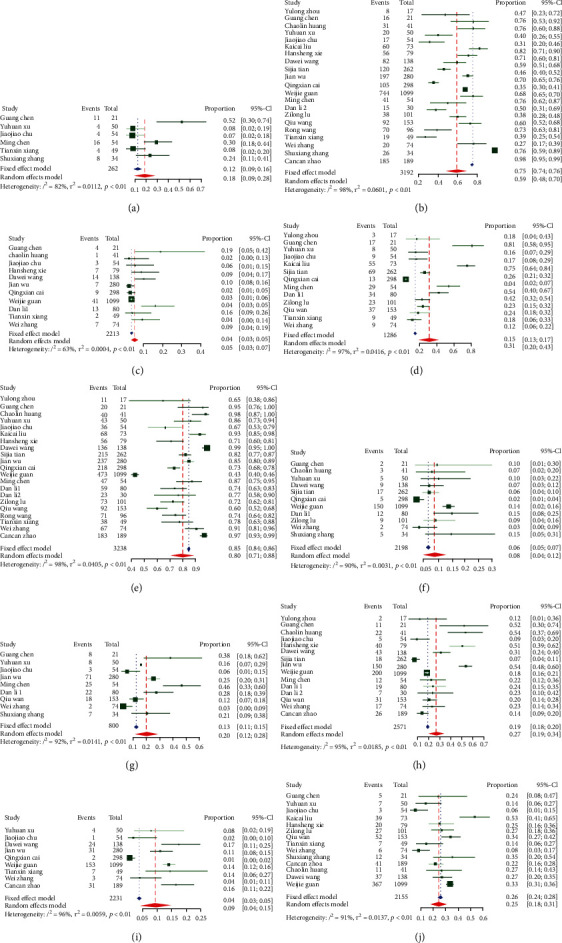
Prevalence of symptoms: (a) chest tightness; (b) cough; (c) diarrhea; (d) fatigue; (e) fever; (f) headache; (g) myalgia; (h) shortness of breath; (i) sore throat; (j) sputum production.

**Figure 5:**
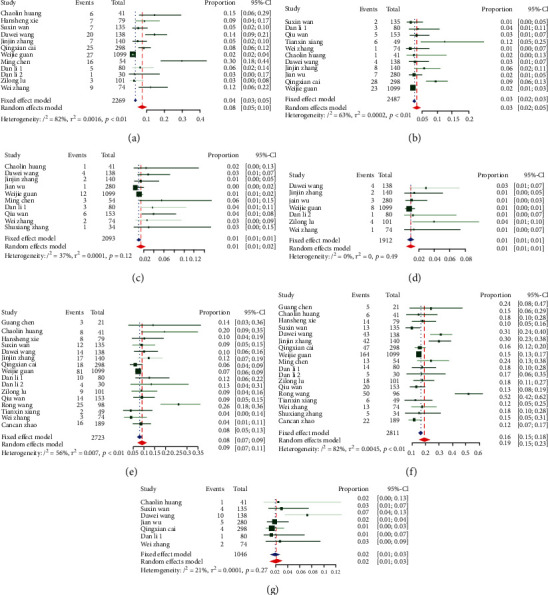
Prevalence of comorbidities: (a) cardiovascular disease; (b) chronic liver disease; (c) chronic respiratory system diseases; (d) chronic renal diseases; (e) diabetes; (f) hypertension; (g) malignancy.

A meta-analysis of all the studies (Table 2) revealed that the most prevalent clinical symptoms were fever (80%, 95% CI: 71-88%) and cough (59%, 95% CI: 48-70%); the most prevalent clinical chronic comorbidity was hypertension (19%, 95% CI: 15-23%), followed by diabetes (9%, 95% CI: 7-11%) and cardiovascular disease (8%, 95% CI: 5-10%). The forest maps with Cochran's *Q* and *I*^2^ index are shown in Figures 4 and 5.

Table 3 presents the diagnostic test results of comorbidities and clinical symptoms for severe patients. The highest positive likelihood ratios of clinical features were found to be chronic respiratory system diseases, chronic renal diseases, chest tightness, cardiovascular disease, and shortness of breath and some other comorbidities in order, with high DOR, high specificity, and low sensitivity, which means that patients with these characteristics are more likely to become severe illness. No measured clinical feature has a low negative likelihood ratio, so based on existing research, the possibility of severe illness cannot be ruled out based on clinical features. It was found that clinical features including fever, cough, and headache, as well as some comorbidities (e.g., hypertension, malignancy), have little warning value. The diagnostic test results for each indicator in each study are provided in Table S1.

COVID-19 patients had normal or decreased white blood cell counts, decreased lymphocyte counts, and increased C-reactive protein. By comparing the laboratory examination of severe patients and nonsevere in the included studies, it was clear that the biochemical indicators of the two groups of patients were significantly different. The most obvious was C-reactive protein, and the ratio of C-reactive protein increased by 1.66 at least, followed by increased ratio of neutrophils to lymphocytes and erythrocyte sedimentation rate in severe patients compared to nonsevere (Table 4).

**Figure 6:**
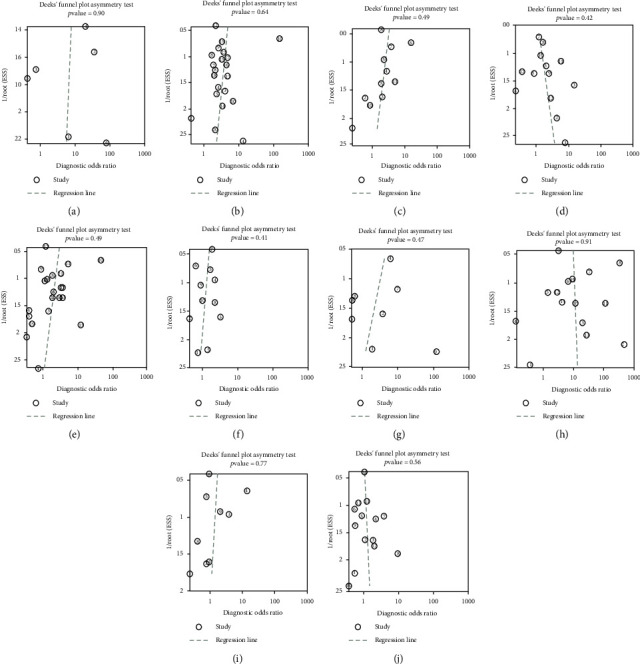
Publication bias analysis: (a) chest tightness; (b) cough; (c) diarrhea; (d) fatigue; (e) fever; (f) headache; (g) myalgia; (h) shortness of breath; (i) sore throat; (j) sputum production.

**Figure 7:**
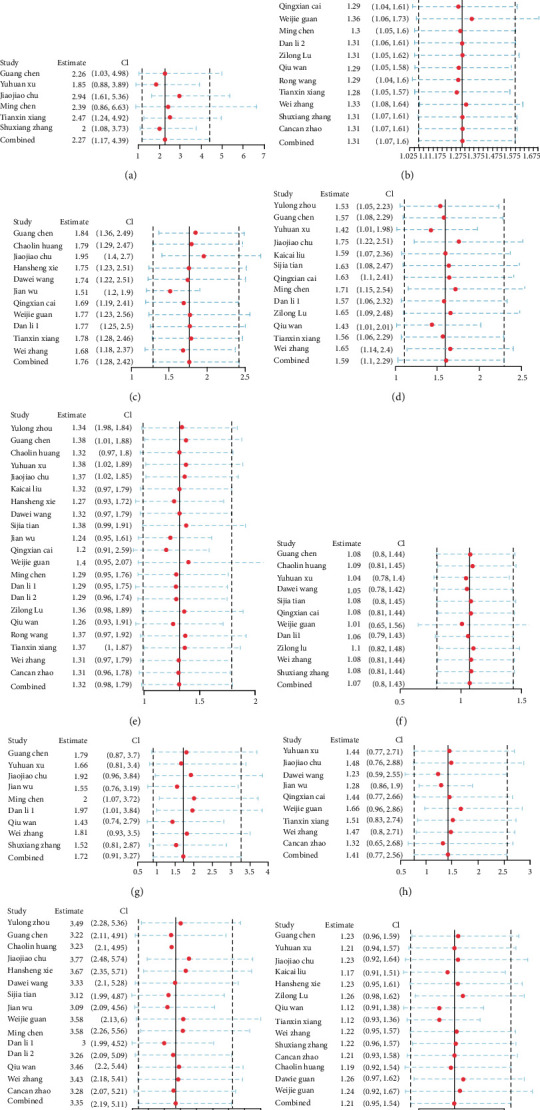
Sensitivity analysis of symptoms: (a) chest tightness; (b) cough; (c) diarrhea; (d) fatigue; (e) fever; (f) headache; (g) myalgia; (h) sore throat; (i) shortness of breath; (j) sputum production.

**Figure 8:**
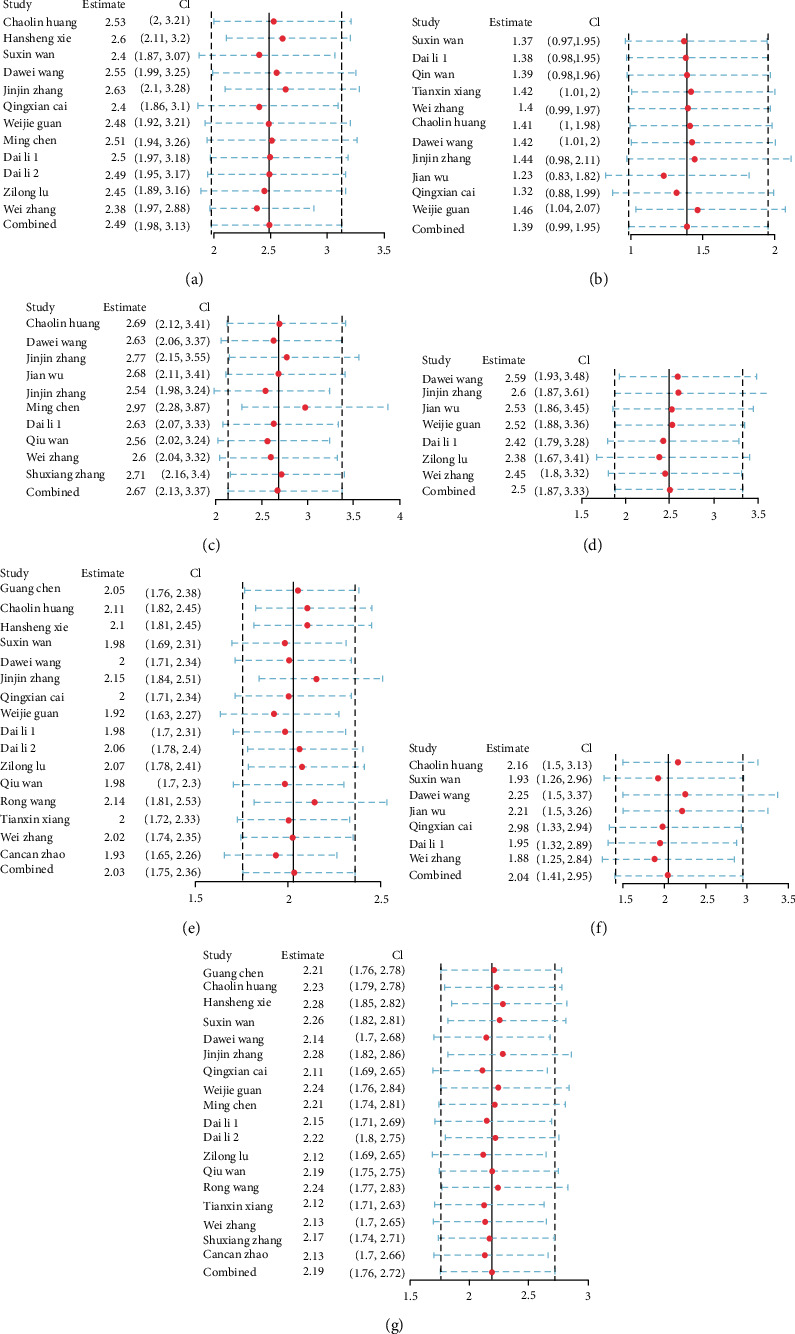
Sensitivity analysis of comorbidities: (a) cardiovascular disease; (b) chronic liver disease; (c) chronic renal diseases; (d) chronic respiratory system diseases; (e) diabetes; (f) hypertension; (g) malignancy.

Publication bias analysis is presented in the form of Deek's funnel plot asymmetry test (Figure 6). The *p* values of the test results of all indicators are all greater than 0.05, so there is no publication bias. Sensitivity analysis is presented in the form of forest maps (Figures 7 and 8), all studies are within reasonable intervals, and sensitivity is acceptable.

## 4. Discussion

COVID-19 is currently experiencing an outbreak period, and the number of patients has continued to rise. The total number of patients has far exceeded that of SARS or MERS. Genome-wide sequencing and phylogenetic tree analysis suggest that COVID-19 is a distinct branch of *β* coronavirus associated with SARS (SARS-CoV) and MERS (MERS-CoV). COVID-19 is characteristic of the coronavirus family and is classified as a *β* coronavirus 2*β* spectrum. Research findings demonstrate that COVID-19 has a similar gene sequence to that of SARS and that they have a common origin (bats) and common intracellular receptors (ACE2) [27]. Thus, its symptoms are also similar to those of SARS, often manifesting as a fever, shortness of breath, cough, or breathing difficulty, and, in severe cases, pneumonia or even death may occur [28]. A study by Guan et al. found that only 43.1 percent of patients with COVID-19 presented with a fever when they were admitted to the hospital, and more developed a fever in the hospital [29]. In contrast, SARS and MERS patients almost all present fevers when they are diagnosed, and only 1-2% do not present a fever [30]. This means that if the examination of suspected COVID-19 cases for the prevention and control of the epidemic is carried out only by measuring body temperature, huge amounts of infected patients without fevers may be omitted. Therefore, COVID-19 is more concealed and more contagious than SARS, and this outbreak will be more difficult to deal with than SARS or MERS. Many mild patients who have rapidly progressed to severe illness have not yet undergone effective targeted therapy for COVID-19. Therefore, understanding the development of the disease is particularly important for reducing the critical rate.

The present study revealed that chest tightness with a positive likelihood ratio 3.8 (1.44, 10.05) and shortness of breath with a positive likelihood ratio 3.18 (2.24, 4.51) have strong correlations with severe patients. Chest tightness and shortness of breath are a sign that the disease seriously affects the respiratory system. Diarrhea also has a relatively high positive likelihood ratio 2.04 (1.38, 3.04), which may be related to severe illness. The main reason for this may be that the throat is connected to the esophagus, and the virus may enter the digestive tract through the throat in severe patients by infecting intestinal epithelial cells and activating the intestinal immune response, thereby stimulating the gastrointestinal tract and causing diarrhea. It has also been recently reported that the disease may be transmitted via the fecal-oral pathway, and intestinal syndrome may be a marker of high viral load. A meta-analysis of the selected data also revealed that the prevalence of hypertension is 19%, 95% CI: 15-23%, followed by diabetes (9%, 95% CI: 7-11%) and cardiovascular disease (8%, 95% CI: 5-10%). And compared to patients with no comorbidities, severe cases are significantly correlated with chronic respiratory system diseases (pooled positive likelihood 6.07, 95% CI: 3.12, 11.82), chronic renal disease (4.79, 2.04-11.25), and cardiovascular disease (3.45, 2.19-5.44). The chronic conditions that influence the severity of COVID-19, such as chronic respiratory system diseases and chronic renal disease, were also reported to affect other coronavirus-infected pneumonia, such as MERS and SARS [31]. Therefore, the roles of comorbidities in severe COVID-19 cases were further investigated. Most healthy young people are mild patients because their lung epithelial cells are in good condition, and their natural response to virus invasion is rapid and effective. Their immune cells are intact and well-functioning, and even after COVID-19 infection and relatively few lower respiratory tract infections, they may not have clinical symptoms or only symptoms that are very mild. In the condition of chronic respiratory disease, especially COPD, lower respiratory tract infections, both acute and chronic, occur with increased frequency [32]. As chronic respiratory disease has a great influence on the clinical course of COVID-19, it can be regarded as a major comorbidity of COVID-19. A large number of immune cells are stimulated by the COVID-19 virus to be recruited to the alveolar site, thus releasing a large number of inflammatory cytokines (known medically as cytokine storms) [33]. These inflammatory factors act on the alveolar-vascular membrane, destroy its integrity, greatly increase its permeability, and result in a large amount of blood in the alveoli, thus leading to human hypoxia and exacerbating respiratory disease like COPD [31]. In addition, since the use of inhaled corticosteroids in COPD patients is positively correlated with the occurrence of pneumonia, it has received considerable attention in recent years [34]. Several studies have determined the role of COPD in the pathogenesis of viral infectious pneumonia [35, 36]. It can also reasonably explain why chronic respiratory diseases (mainly COPD) are more likely to cause disease deterioration. Studies have found that, compared with non-HIV-infected patients, patients with chronic respiratory disease are more likely to become ill with HIV infection [37]. Therefore, the overuse of hormone drugs makes patients with chronic respiratory diseases more susceptible to viruses such as COVID-19.

Chronic metabolic diseases such as diabetes, chronic cardiovascular diseases, chronic renal diseases, and related conditions can be etiologically linked to the pathogenesis of COVID-19. Existing research has found that COVID-19 may be a self-limiting disease, and the body can clear the virus on its own if it has sufficient immunity. These comorbidities are known as “downregulators” of the innate immune system of the host during the progress of the body's response to pathogenesis. Chronic diseases have shown similar potential in causing infectious disorders of the immune system [38, 39]. For example, diabetes decreases the synthesis of proinflammatory cytokines, interleukins, and their downstream acute phase reactant [40]. Metabolic disorders also influence macrophage and lymphocyte functions which result in a decline of immunity system and may finally cause patients to be more susceptible to COVID-19. In elderly patients with cardiovascular disease, diabetes, or other basic diseases, the immune system function is weaker, their ability to resist the virus is worse, and they are more vulnerable to infection by COVID-19. Therefore, improving the basic conditions of the body, such as lowering blood sugar, is likely to control the deterioration of COVID-19 and reduce mortality.

In addition, the patient's laboratory examination is also expected to be used as an important indicator to predict the severity of the patient's disease. When the patient's C-reactive protein, ratio of neutrophils to lymphocytes, and erythrocyte sedimentation rate increased, the patient is more likely to progress to severe disease and require critical care. C-reactive protein is an indicator of the degree of infection. When it increases violently, it indicates that the patient is in serious condition and has a high probability of worsening into severe. The same is true for other relevant indicators, reflecting the progress of the disease infection. Therefore, laboratory examination must not be ignored, so that clinicians can detect patients' deteriorating progress earlier and take effective treatment in time.

In conclusion, this study has found that those who have been diagnosed with chronic conditions especially chronic respiratory disease are more susceptible to COVID-19, and we should be more alert to the serious development of COVID-19 in patients with chest tightness, shortness of breath, and diarrhea and also the neglected laboratory examination like increased C-reactive protein, ratio of neutrophils to lymphocytes, and erythrocyte sedimentation rate. Our research could be used to properly monitor and predict the serious progression in patients with COVID-19 to reduce mortality.

The present study has several limitations. First, the largest sample in this study occupies 1,099/3,547 (31.0%) of the total sample, which is a large proportion and may have a greater impact on the overall conclusion. Second, different articles have different definitions of severe patients, which may cause deviations in result. Third, some of the articles have overlapping definitions of certain test indicators, affecting the data and results. Fourth, it is not clear whether the indicators in some studies were determined at the time of admission, and the time order of the occurrence of symptoms and the severity of the disease may affect the results. Fifth, the end points of the patients in the including studies were unclear, and the cut-off point was the publication time of the article. It was difficult to determine whether the future mutual conversion between mild and severe cases would affect the results. Nevertheless, the conclusions drawn so far are still of great significance to the treatment of COVID-19 today.

## Figures and Tables

**Table 1:** The basic information of the included studies.

Number	Study	Year	*n*	Country	Indicators included in the study
1	Yulong Zhou	2020	17	China	Cough, fatigue, fever, shortness of breath
2	Guang Chen	2020	21	China	Chest tightness, cough, diabetes, diarrhea, fatigue, fever, headache, hypertension, myalgia, shortness of breath, sputum production
3	Chaolin Huang	2020	41	China	Cardiovascular disease, chronic liver disease, chronic respiratory system diseases, cough, diabetes, diarrhea, fever, headache, hypertension, malignancy, shortness of breath, sputum production
4	Yuhuan Xu	2020	50	China	Chest tightness, cough, fatigue, fever, headache, myalgia, sore throat, sputum production
5	Jiaojiao Chu	2020	54	China	Chest tightness, cough, diarrhea, fatigue, fever, myalgia, shortness of breath, sore throat, sputum production
6	Kaicai Liu	2020	73	China	Cough, fatigue, fever, sputum production
7	Hansheng Xie	2020	79	China	Cardiovascular disease, cough, diabetes, diarrhea, fever, hypertension, shortness of breath, sputum production
8	Suxin Wan	2020	135	China	Cardiovascular disease, chronic liver disease, diabetes, hypertension, malignancy
9	Dawei Wang	2020	138	China	Cardiovascular disease, chronic liver disease, chronic renal diseases, chronic respiratory system diseases, cough, diabetes, diarrhea, fever, headache, hypertension, malignancy, shortness of breath, sore throat, sputum production
10	Jinjin Zhang	2020	140	China	Cardiovascular disease, chronic liver disease, chronic renal diseases, chronic respiratory system diseases, diabetes, hypertension
11	Sijia Tian	2020	262	China	Cough, fatigue, fever, headache, shortness of breath
12	Jian Wu	2020	280	China	Chronic liver disease, chronic renal diseases, chronic respiratory system diseases, cough, diarrhea, fever, malignancy, myalgia, shortness of breath, sore throat
13	Qingxian Cai	2020	298	China	Cardiovascular disease, chronic liver disease, cough, diabetes, diarrhea, fatigue, fever, headache, hypertension, malignancy, sore throat
14	Weijie Guan	2020	1099	China	Cardiovascular disease, chronic liver disease, chronic renal diseases, chronic respiratory system diseases, cough, diabetes, diarrhea, fever, headache, hypertension, shortness of breath, sore throat, sputum production
15	Ming Chen	2020	54	China	Cardiovascular disease, chest tightness, chronic respiratory system diseases, cough, fatigue, fever, hypertension, myalgia, shortness of breath
16	Dan Li 1	2020	80	China	Cardiovascular disease, chronic liver disease, chronic renal diseases, chronic respiratory system diseases, diabetes, diarrhea, fatigue, fever, headache, hypertension, malignancy, myalgia, shortness of breath
17	Dan Li 2	2020	30	China	Cardiovascular disease, cough, diabetes, fever, hypertension, shortness of breath
18	Zilong Lu	2020	101	China	Cardiovascular disease, chronic renal diseases, cough, diabetes, fatigue, fever, headache, hypertension, sputum production
19	Qiu Wan	2020	153	China	Chronic liver disease, chronic respiratory system diseases, cough, diabetes, fatigue, fever, hypertension, myalgia, shortness of breath, sputum production
20	Rong Wang	2020	96	China	Cough, diabetes, fever, hypertension
21	Tianxin Xiang	2020	49	China	Chest tightness, chronic liver disease, cough, diabetes, diarrhea, fatigue, fever, hypertension, sore throat, sputum production
22	Wei Zhang	2020	74	China	Cardiovascular disease, chronic liver disease, chronic renal diseases, chronic respiratory system diseases, cough, diabetes, diarrhea, fatigue, fever, headache, hypertension, malignancy, myalgia, shortness of breath, sore throat, sputum production, chest tightness
23	Shuxiang Zhang	2020	34	China	Chronic respiratory system diseases, cough, headache, hypertension, myalgia, sputum production
24	Cancan Zhao	2020	189	China	Cough, diabetes, fever, hypertension, shortness of breath, sore throat, sputum production

**Table 2:** Baseline characteristics of patients in the included studies and meta-analysis.

Characteristics	Prevalence (%)	95% CI
Male	54	50-57
Severe	30	25-35
Symptom		
Chest tightness	18	9-28
Cough	59	48-70
Diarrhea	5	3-7
Fatigue	31	20-43
Fever	80	71-88
Headache	8	4-12
Myalgia	20	12-28
Shortness of breath	27	19-34
Sore throat	9	4-15
Sputum production	25	18-31
Comorbidities		
Cardiovascular disease	8	5-10
Chronic liver disease	3	2-5
Chronic renal diseases	1	1-1
Chronic respiratory system diseases	1	1-1
Diabetes	9	7-11
Hypertension	19	15-23
Malignancy	2	1-3

**Table 3:** Sensitivity, specificity, positive likelihood ratio, negative likelihood ratio, and diagnostic ratio of each clinical feature.

	Pooled sensitivity	Pooled specificity	Pooled LR+	Pooled LR-	Pooled DOR
Symptom					
Chest tightness	0.31 (0.23, 0.41)	0.92 (0.86, 0.96)	3.8 (1.44, 10.05)	0.71 (0.46, 1.1)	6.73 (1.44, 31.48)
Cough	0.69 (0.66, 0.72)	0.41 (0.39, 0.43)	1.21 (1.06, 1.37)	0.81 (0.7, 0.94)	1.6 (1.2, 2.1)
Diarrhea	0.09 (0.06, 0.11)	0.96 (0.95, 0.97)	2.04 (1.38, 3.04)	0.96 (0.93, 0.99)	2.21 (1.43, 3.42)
Fatigue	0.34 (0.29, 0.39)	0.79 (0.76, 0.81)	1.39 (1.04, 1.87)	0.88 (0.76, 1.03)	1.81 (1.07, 3.06)
Fever	0.78 (0.75, 0.81)	0.35 (0.33, 0.37)	1.09 (1.03, 1.17)	0.72 (0.53, 0.99)	1.62 (1.1, 2.4)
Headache	0.09 (0.07, 0.13)	0.9 (0.88, 0.91)	1.07 (0.78, 1.46)	1.01 (0.99, 1.03)	1.06 (0.74, 1.53)
Myalgia	0.34 (0.28, 0.41)	0.86 (0.82, 0.88)	2 (0.95, 4.22)	0.86 (0.63, 1.16)	2.37 (0.84, 6.65)
Shortness of breath	0.5 (0.46, 0.54)	0.84 (0.83, 0.86)	3.18 (2.24, 4.51)	0.65 (0.51, 0.82)	7.6 (3.59, 16.07)
Sore throat	0.15 (0.12, 0.19)	0.9 (0.88, 0.91)	1.69 (0.81, 3.55)	0.95 (0.87, 1.05)	1.77 (0.73, 4.31)
Sputum production	0.3 (0.26, 0.34)	0.71 (0.68, 0.73)	1.22 (0.95, 1.57)	0.98 (0.92, 1.06)	1.28 (0.9, 1.84)
Comorbidities					
Cardiovascular disease	0.14 (0.12, 0.18)	0.97 (0.96, 0.98)	3.45 (2.19, 5.44)	0.87 (0.82, 0.95)	4.18 (2.44, 7.16)
Chronic liver disease	0.04 (0.02, 0.06)	0.96 (0.96, 0.97)	1.36 (0.82, 2.26)	1 (0.98, 1.02)	1.37 (0.8, 2.36)
Chronic renal disease	0.04 (0.02, 0.06)	1 (0.99, 1)	4.79 (2.04, 11.25)	0.98 (0.95, 1)	4.98 (2.08, 11.91)
Chronic respiratory system diseases	0.05 (0.03, 0.08)	0.99 (0.99, 1)	6.07 (3.12, 11.82)	0.97 (0.95, 0.99)	6.53 (3.26, 13.06)
Diabetes	0.19 (0.16, 0.22)	0.94 (0.93, 0.95)	2.82 (1.95, 4.1)	0.89 (0.84, 0.95)	3.25 (2.11, 5.03)
Hypertension	0.34 (0.3, 0.38)	0.87 (0.85, 0.88)	2.53 (1.89, 3.4)	0.78 (0.61, 0.88)	3.47 (2.3, 5.21)
Malignancy	0.05 (0.03, 0.09)	0.98 (0.97, 0.99)	2.83 (1.34, 5.98)	0.98 (0.95, 1)	3.03 (1.38, 6.65)

**Table 4:** The ratio of severe to nonsevere patients in laboratory tests.

Study	Yulong Zhou	Guang Chen	Chaolin Huang	Hansheng Xie	Suxin Wan	Dawei Wang	Jinjin Zhang	Jian Wu	Qingxian Cai	Weijie Guan	Dan Li 1	Dan Li 2	Zilong Lu	Qiu Wan	Tianxin Xiang
Blood leukocyte count	0.62	1.96	1.98	1.12	0.95	1.53	1.18	0.68	0.99	1.32	1.46	1.54	1.19	0.64	2.07
Neutrophil count		2.58	2.41	1.03	1.14	1.70		0.71	1.11			2.17	1.66		2.62
Lymphocyte count	0.53	0.64	0.40	0.92	0.67	0.89	0.88	0.38	0.73	1.25	0.65	0.47	0.50		0.57
Neutrophil/lymphocyte		4.06	6.02	1.12	1.71	1.92		1.85	1.51			4.63	3.30		4.59
Platelet count		1.02	1.32		0.86	0.86		0.44		1.25	0.92	1.20	0.78		1.09
Haemoglobin level		1.02	0.93		0.97			1.01		1.05	0.88	1.00			1.01
Monocyte						1.00		1.25							1.06
Eosinophils							0.50		0.50						35.00
Albumin	1.02	0.84	0.80		0.72			0.90			0.89	0.80			0.84
Aspartate aminotransferase		2.11	1.29	1.25	1.50	1.79		1.00	1.38		1.41	1.41	0.98		1.52
Alanine aminotransferase		2.35	1.81	1.22	1.23	1.52		1.20	1.34		1.49	2.04	0.73		1.55
D-dimer	0.97	20.50	4.80	1.04	2.00	2.49	2.00	15.00	1.56		2.85	34.00		1.88	20.96
Sodium			0.99		0.98					1.00					0.98
Potassium			1.12		0.95					1.03					1.03
Chloride										1.00					1.01
Prothrombin time		1.05	1.14		1.05	1.02		1.00			1.11	0.93			1.12
Activated partial thromboplastin time		0.81	0.95		1.12	0.96		0.95				0.94			1.31
Total bilirubin		1.37	1.30	0.91	1.14	1.24		1.02	1.03			1.04			1.53
Creatinine		1.24	1.08	0.98	0.96	1.13		1.09	1.18		1.05	0.99	1.19		1.01
Creatine kinase		1.95	0.99		1.44	1.17	0.80	1.13	1.35		1.33	0.91	1.11		1.41
Lactate dehydrogenase	0.87	2.42	1.42		1.46	2.05		1.28	1.79		1.62	1.58	1.29	1.62	1.97
C-reactive protein	2.67	2.63		3.20	11.82		1.66	3.09	3.94		3.97	5.63	2.22	3.61	4.98
ALP				0.94					0.95						5.10
Hypersensitive troponin I			0.94												
Procalcitonin		5.00	1.00		2.75		2.00	1.15	0.80		11.75			2.00	4.00
Blood urea nitrogen		1.83				1.23		1.10	1.35			1.29	1.54		1.33
Erythrocyte sedimentation rate				1.26				1.17	1.88		3.28				1.83

## Supplementary Materials

Supplementary 1 Table S1: the diagnostic test results for each indicator in each study are provided.

Supplementary 2 PRISMA-P (Preferred Reporting Items for Systematic review and Meta-Analysis Protocols) 2015 checklist: recommended items to address in a systematic review protocol.
