# Supplementary material for: Clinical Features and Laboratory Examination to Identify Severe Patients with COVID-19: A Systematic Review and Meta-Analysis
Source: Biomed Res Int. 2021 Nov 15;2021:6671291. doi: 10.1155/2021/6671291 (PMC8593588; doi:10.1155/2021/6671291)
Supplement: Supplementary 1 — Table S1: the diagnostic test results for each indicator in each study are provided. [file 6671291.f1.pdf]

	Disease	Study	Estimate	95%CI
2	cardiacdisease	Chaolin Huang	2.53	(2,3.21)
3	cardiacdisease	Hansheng Xie	2.6	(2.11,3.2)
4	cardiacdisease	Suxin Wan	2.4	(1.87,3.07)
5	cardiacdisease	Dawei Wang	2.55	(1.99,3.25)
6	cardiacdisease	Jinjin Zhang	2.63	(2.1,3.28)
7	cardiacdisease	Qingxian Cai	2.4	(1.86,3.1)
8	cardiacdisease	Weijie Guan	2.48	(1.92,3.21)
9	cardiacdisease	Ming Chen	2.51	(1.94,3.26)
10	cardiacdisease	Dan Li 1	2.5	(1.97,3.18)
11	cardiacdisease	Dan Li 2	2.49	(1.95,3.17)
12	cardiacdisease	Zilong Lu	2.45	(1.89,3.16)
13	cardiacdisease	Wei Zhang	2.38	(1.97,2.88)
14	cardiacdisease	Combined	2.49	(1.98,3.13)
15			NA	(NA,NA)
16	chesttightness	Guang Chen	2.26	(1.03,4.98)
17	chesttightness	Yuhuan Xu	1.85	(0.88,3.89)
18	chesttightness	Jiaojiao Chu	2.94	(1.61,5.36)
19	chesttightness	Ming Chen	2.39	(0.86,6.63)
20	chesttightness	Tianxin Xiang	2.47	(1.24,4.92)
21	chesttightness	Shuxiang Zhang	2	(1.08,3.73)
22	chesttightness	Combined	2.27	(1.17,4.39)
23		--------------	NA	(NA,NA)
24	chronicliverdisease	Suxin Wan	1.37	(0.97,1.95)
25	chronicliverdisease	Dan Li 1	1.38	(0.98,1.95)
26	chronicliverdisease	Qiu Wan	1.39	(0.98,1.96)
27	chronicliverdisease	Tianxin Xiang	1.42	(1.01,2)
28	chronicliverdisease	Wei Zhang	1.4	(0.99,1.97)
29	chronicliverdisease	Chaolin Huang	1.41	(1,1.98)
30	chronicliverdisease	Dawei Wang	1.42	(1.01,2)
31	chronicliverdisease	Jinjin Zhang	1.44	(0.98,2.11)
32	chronicliverdisease	Jian Wu	1.23	(0.83,1.82)
33	chronicliverdisease	Qingxian Cai	1.32	(0.88,1.99)
34	chronicliverdisease	Weijie Guan	1.46	(1.04,2.07)
35	chronicliverdisease	Combined	1.39	(0.99,1.95)
36		--------------	NA	(NA,NA)
37	Chronicreldiseases	Dawei Wang	2.59	(1.93,3.48)
38	Chronicreldiseases	Jinjin Zhang	2.6	(1.87,3.61)
39	Chronicreldiseases	Jian Wu	2.53	(1.86,3.45)
40	Chronicreldiseases	Weijie Guan	2.52	(1.88,3.36)
41	Chronicreldiseases	Dan Li 1	2.42	(1.79,3.28)
42	Chronicreldiseases	Zilong Lu	2.38	(1.67,3.41)
43	Chronicreldiseases	Wei Zhang	2.45	(1.8,3.32)
44	Chronicreldiseases	Combined	2.5	(1.87,3.33)
45		--------------	NA	(NA,NA)
46	ChronicRespiratorys	Chaolin Huang	2.69	(2.12,3.41)
47	ChronicRespiratorys	Dawei Wang	2.63	(2.06,3.37)
48	ChronicRespiratorys	Jinjin Zhang	2.77	(2.15,3.55)
49	ChronicRespiratorys	Jian Wu	2.68	(2.11,3.41)
50	ChronicRespiratorys	Weijie Guan	2.54	(1.98,3.24)
51	ChronicRespiratorys	Ming Chen	2.97	(2.28,3.87)
52	ChronicRespiratorys	Dan Li 1	2.63	(2.07,3.33)
53	ChronicRespiratorys	Qiu Wan	2.56	(2.02,3.24)
54	ChronicRespiratorys	Wei Zhang	2.6	(2.04,3.32)
55	ChronicRespiratorys	Shuxiang Zhang	2.71	(2.16,3.4)
56	ChronicRespiratorys	Combined	2.67	(2.13,3.37)
57		--------------	NA	(NA,NA)
58	Cough	Yulong Zhou	1.29	(1.06,1.58)

59	Cough	Guang Chen	1.35	(1.12,1.63)
60	Cough	Chaolin Huang	1.3	(1.06,1.6)
61	Cough	Yuhuan Xu	1.32	(1.07,1.62)
62	Cough	Jiaojiao Chu	1.35	(1.09,1.66)
63	Cough	Kaicai Liu	1.32	(1.07,1.63)
64	Cough	Hansheng Xie	1.33	(1.08,1.65)
65	Cough	Dawei Wang	1.35	(1.09,1.67)
66	Cough	Sijia Tian	1.31	(1.05,1.63)
67	Cough	Jian Wu	1.23	(1.08,1.4)
68	Cough	Qingxian Cai	1.29	(1.04,1.61)
69	Cough	Weijie Guan	1.36	(1.06,1.73)
70	Cough	Ming Chen	1.3	(1.05,1.6)
71	Cough	Dan Li 2	1.31	(1.06,1.61)
72	Cough	Zilong Lu	1.31	(1.05,1.62)
73	Cough	Qiu Wan	1.29	(1.05,1.58)
74	Cough	Rong Wang	1.29	(1.04,1.6)
75	Cough	Tianxin Xiang	1.28	(1.05,1.57)
76	Cough	Wei Zhang	1.33	(1.08,1.64)
77	Cough	Shuxiang Zhang	1.31	(1.07,1.61)
78	Cough	Cancan Zhao	1.31	(1.07,1.61)
79	Cough	Combined	1.31	(1.07,1.6)
80		--------------	NA	(NA,NA)
81	Diabetes	Guang Chen	2.05	(1.76,2.38)
82	Diabetes	Chaolin Huang	2.11	(1.82,2.45)
83	Diabetes	Hansheng Xie	2.1	(1.81,2.45)
84	Diabetes	Suxin Wan	1.98	(1.69,2.31)
85	Diabetes	Dawei Wang	2	(1.71,2.34)
86	Diabetes	Jinjin Zhang	2.15	(1.84,2.51)
87	Diabetes	Qingxian Cai	2	(1.71,2.34)
88	Diabetes	Weijie Guan	1.92	(1.63,2.27)
89	Diabetes	Dan Li 1	1.98	(1.7,2.31)
90	Diabetes	Dan Li 2	2.06	(1.78,2.4)
91	Diabetes	Zilong Lu	2.07	(1.78,2.41)
92	Diabetes	Qiu Wan	1.98	(1.7,2.3)
93	Diabetes	Rong Wang	2.14	(1.81,2.53)
94	Diabetes	Tianxin Xiang	2	(1.72,2.33)
95	Diabetes	Wei Zhang	2.02	(1.74,2.35)
96	Diabetes	Cancan Zhao	1.93	(1.65,2.26)
97	Diabetes	Combined	2.03	(1.75,2.36)
98		--------------	NA	(NA,NA)
99	Diarrhea	Guang Chen	1.84	(1.36,2.49)
100	Diarrhea	Chaolin Huang	1.79	(1.29,2.47)
101	Diarrhea	Jiaojiao Chu	1.95	(1.4,2.7)
102	Diarrhea	Hansheng Xie	1.75	(1.23,2.51)
103	Diarrhea	Dawei Wang	1.74	(1.22,2.51)
104	Diarrhea	Jian Wu	1.51	(1.2,1.9)
105	Diarrhea	Qingxian Cai	1.69	(1.19,2.41)
106	Diarrhea	Weijie Guan	1.77	(1.23,2.56)
107	Diarrhea	Dan Li 1	1.77	(1.25,2.5)
108	Diarrhea	Tianxin Xiang	1.78	(1.28,2.46)
109	Diarrhea	Wei Zhang	1.68	(1.18,2.37)
110	Diarrhea	Combined	1.76	(1.28,2.42)
111		--------------	NA	(NA,NA)
112	Fatigue	Yulong Zhou	1.53	(1.05,2.23)
113	Fatigue	Guang Chen	1.57	(1.08,2.29)
114	Fatigue	Yuhuan Xu	1.42	(1.01,1.98)
115	Fatigue	Jiaojiao Chu	1.75	(1.22,2.51)
116	Fatigue	Kaicai Liu	1.59	(1.07,2.36)

117	Fatigue	Sijia Tian	1.63 (1.08,2.47)
118	Fatigue	Qingxian Cai	1.63 (1.1,2.41)
119	Fatigue	Ming Chen	1.71 (1.15,2.54)
120	Fatigue	Dan Li 1	1.57 (1.06,2.32)
121	Fatigue	Zilong Lu	1.65 (1.09,2.48)
122	Fatigue	Qiu Wan	1.43 (1.01,2.01)
123	Fatigue	Tianxin Xiang	1.56 (1.06,2.29)
124	Fatigue	Wei Zhang	1.65 (1.14,2.4)
125	Fatigue	Combined	1.59 (1.1,2.29)
126		--------------NA	(NA,NA)
127	Fever	Yulong Zhou	1.34 (0.98,1.84)
128	Fever	Guang Chen	1.38 (1.01,1.88)
129	Fever	Chaolin Huang	1.32 (0.97,1.8)
130	Fever	Yuhuan Xu	1.38 (1.02,1.89)
131	Fever	Jiaojiao Chu	1.37 (1.02,1.85)
132	Fever	Kaicai Liu	1.32 (0.97,1.79)
133	Fever	Hansheng Xie	1.27 (0.93,1.72)
134	Fever	Dawei Wang	1.32 (0.97,1.79)
135	Fever	Sijia Tian	1.38 (0.99,1.91)
136	Fever	Jian Wu	1.24 (0.95,1.61)
137	Fever	Qingxian Cai	1.2 (0.91,1.59)
138	Fever	Weijie Guan	1.4 (0.95,2.07)
139	Fever	Ming Chen	1.29 (0.95,1.76)
140	Fever	Dan Li 1	1.29 (0.95,1.75)
141	Fever	Dan Li 2	1.29 (0.96,1.74)
142	Fever	Zilong Lu	1.36 (0.98,1.89)
143	Fever	Qiu Wan	1.26 (0.93,1.71)
144	Fever	Rong Wang	1.37 (0.97,1.92)
145	Fever	Tianxin Xiang	1.37 (1,1.87)
146	Fever	Wei Zhang	1.31 (0.97,1.79)
147	Fever	Cancan Zhao	1.31 (0.96,1.78)
148	Fever	Combined	1.32 (0.98,1.79)
149		--------------NA	(NA,NA)
150	Headache	Guang Chen	1.08 (0.8,1.44)
151	Headache	Chaolin Huang	1.09 (0.81,1.45)
152	Headache	Yuhuan Xu	1.04 (0.78,1.4)
153	Headache	Dawei Wang	1.05 (0.78,1.42)
154	Headache	Sijia Tian	1.08 (0.8,1.45)
155	Headache	Qingxian Cai	1.08 (0.81,1.44)
156	Headache	Weijie Guan	1.01 (0.65,1.56)
157	Headache	Dan Li 1	1.06 (0.79,1.43)
158	Headache	Zilong Lu	1.1 (0.82,1.48)
159	Headache	Wei Zhang	1.08 (0.81,1.44)
160	Headache	Shuxiang Zhang	1.08 (0.81,1.44)
161	Headache	Combined	1.07 (0.8,1.43)
162		--------------NA	(NA,NA)
163	Hypertension	Guang Chen	2.21 (1.76,2.78)
164	Hypertension	Chaolin Huang	2.23 (1.79,2.78)
165	Hypertension	Hansheng Xie	2.28 (1.85,2.82)
166	Hypertension	Suxin Wan	2.26 (1.82,2.81)
167	Hypertension	Dawei Wang	2.14 (1.7,2.68)
168	Hypertension	Jinjin Zhang	2.28 (1.82,2.86)
169	Hypertension	Qingxian Cai	2.11 (1.69,2.65)
170	Hypertension	Weijie Guan	2.24 (1.76,2.84)
171	Hypertension	Ming Chen	2.21 (1.74,2.81)
172	Hypertension	Dan Li 1	2.15 (1.71,2.69)
173	Hypertension	Dan Li 2	2.22 (1.8,2.75)
174	Hypertension	Zilong Lu	2.12 (1.69,2.65)

175	Hypertension	Qiu Wan	2.19	(1.75,2.75)
176	Hypertension	Rong Wang	2.24	(1.77,2.83)
177	Hypertension	Tianxin Xiang	2.12	(1.71,2.63)
178	Hypertension	Wei Zhang	2.13	(1.7,2.65)
179	Hypertension	Shuxiang Zhang	2.17	(1.74,2.71)
180	Hypertension	Cancan Zhao	2.13	(1.7,2.66)
181	Hypertension	Combined	2.19	(1.76,2.72)
182		--------------NA		(NA,NA)
183	maligncy	Chaolin Huang	2.16	(1.5,3.13)
184	maligncy	Suxin Wan	1.93	(1.26,2.96)
185	maligncy	Dawei Wang	2.25	(1.5,3.37)
186	maligncy	Jian Wu	2.21	(1.5,3.26)
187	maligncy	Qingxian Cai	1.98	(1.33,2.94)
188	maligncy	Dan Li 1	1.95	(1.32,2.89)
189	maligncy	Wei Zhang	1.88	(1.25,2.84)
190	maligncy	Combined	2.04	(1.41,2.95)
191		--------------NA		(NA,NA)
192	Myalgia	Guang Chen	1.79	(0.87,3.7)
193	Myalgia	Yuhuan Xu	1.66	(0.81,3.4)
194	Myalgia	Jiaojiao Chu	1.92	(0.96,3.84)
195	Myalgia	Jian Wu	1.55	(0.76,3.19)
196	Myalgia	Ming Chen	2	(1.07,3.72)
197	Myalgia	Dan Li 1	1.97	(1.01,3.84)
198	Myalgia	Qiu Wan	1.43	(0.74,2.79)
199	Myalgia	Wei Zhang	1.81	(0.93,3.5)
200	Myalgia	Shuxiang Zhang	1.52	(0.81,2.87)
201	Myalgia	Combined	1.72	(0.91,3.27)
202		--------------NA		(NA,NA)
203	Shortofbreath	Yulong Zhou	3.49	(2.28,5.36)
204	Shortofbreath	Guang Chen	3.22	(2.11,4.91)
205	Shortofbreath	Chaolin Huang	3.23	(2.1,4.95)
206	Shortofbreath	Jiaojiao Chu	3.77	(2.48,5.74)
207	Shortofbreath	Hansheng Xie	3.67	(2.35,5.71)
208	Shortofbreath	Dawei Wang	3.33	(2.1,5.28)
209	Shortofbreath	Sijia Tian	3.12	(1.99,4.87)
210	Shortofbreath	Jian Wu	3.09	(2.09,4.56)
211	Shortofbreath	Weijie Guan	3.58	(2.13,6)
212	Shortofbreath	Ming Chen	3.58	(2.26,5.65)
213	Shortofbreath	Dan Li 1	3	(1.99,4.52)
214	Shortofbreath	Dan Li 2	3.26	(2.09,5.09)
215	Shortofbreath	Qiu Wan	3.46	(2.2,5.44)
216	Shortofbreath	Wei Zhang	3.43	(2.18,5.41)
217	Shortofbreath	Cancan Zhao	3.28	(2.07,5.21)
218	Shortofbreath	Combined	3.35	(2.19,5.11)
219		--------------NA		(NA,NA)
220	Sorethroat	Yuhuan Xu	1.44	(0.77,2.71)
221	Sorethroat	Jiaojiao Chu	1.48	(0.76,2.88)
222	Sorethroat	Dawei Wang	1.23	(0.59,2.55)
223	Sorethroat	Jian Wu	1.28	(0.86,1.9)
224	Sorethroat	Qingxian Cai	1.44	(0.77,2.66)
225	Sorethroat	Weijie Guan	1.66	(0.96,2.86)
226	Sorethroat	Tianxin Xiang	1.51	(0.83,2.74)
227	Sorethroat	Wei Zhang	1.47	(0.8,2.71)
228	Sorethroat	Cancan Zhao	1.32	(0.65,2.68)
229	Sorethroat	Combined	1.41	(0.77,2.56)
230		--------------NA		(NA,NA)
231	Sputumproduction	Guang Chen	1.23	(0.96,1.59)
232	Sputumproduction	Yuhuan Xu	1.21	(0.94,1.57)

233	Sputumproduction	Jiaojiao Chu	1.23 (0.92,1.64)
234	Sputumproduction	Kaicai Liu	1.17 (0.91,1.51)
235	Sputumproduction	Hansheng Xie	1.23 (0.95,1.61)
236	Sputumproduction	Zilong Lu	1.26 (0.98,1.62)
237	Sputumproduction	Qiu Wan	1.12 (0.91,1.38)
238	Sputumproduction	Tianxin Xiang	1.12 (0.93,1.36)
239	Sputumproduction	Wei Zhang	1.22 (0.95,1.57)
240	Sputumproduction	Shuxiang Zhang	1.22 (0.96,1.57)
241	Sputumproduction	Cancan Zhao	1.21 (0.93,1.58)
242	Sputumproduction	Chaolin Huang	1.19 (0.92,1.54)
243	Sputumproduction	Dawei Wang	1.26 (0.97,1.62)
244	Sputumproduction	Weijie Guan	1.24 (0.92,1.67)
245	Sputumproduction	Combined	1.21 (0.95,1.54)
