# Supplementary material for: Clinical Features and Laboratory Examination to Identify Severe Patients with COVID-19: A Systematic Review and Meta-Analysis
Source: Biomed Res Int. 2021 Nov 15;2021:6671291. doi: 10.1155/2021/6671291 (PMC8593588; doi:10.1155/2021/6671291)
Supplement: Supplementary 2 — PRISMA-P (Preferred Reporting Items for Systematic review and Meta-Analysis Protocols) 2015 checklist: recommended items to address in a systematic review protocol. [file 6671291.f2.doc]

**PRISMA-P (Preferred Reporting Items for Systematic review and Meta-Analysis Protocols) 2015 checklist: recommended items to address in a systematic review protocol**

| Section and topic | Item No | Checklist item |
| --- | --- | --- |
| ADMINISTRATIVE INFORMATION | | |
| Title: |  |  |
| Identification | 1 | Clinical Features and Laboratory examination to Identify Severe Patients with COVID-19: A Systematic Review and Meta-analysis |
| Registration | 2 | None |
| Authors: |  |  |
| Contact | 3a | Yan Meng^1^* [mengyan_mmm@163.com](mailto:mengyan_mmm@163.com)  Jinpeng Wang^1^* wangjinpeng324@163.com  Kaicheng Wen^1^ [wenkcdr@163.com](mailto:wenkcdr@163.com)  Wacili Da^1^ [dw_7711@163.com](mailto:dw_7711@163.com)  Keda Yang^1^ [15840075023@163.com](mailto:15840075023@163.com)  Siming Zhou^1^ [zsm18_92@163.com](mailto:zsm18_92@163.com)  Zhengbo Tao^1^ [taozhengbo_spine@163.com](mailto:taozhengbo_spine@163.com)  Hang Liu^3^ [hliu34@mgh.harvard.edu](mailto:hliu34@mgh.harvard.edu)  Lin Tao^1,2^# taolindr@163.com  ^1^The First Hospital of China Medical University, Shenyang 110001, Liaoning, China  ^2^Institute of Health Sciences of China Medical University, Shenyang, 110001, Liaoning, China.  ^3^Ragon Institute of MGH, MIT and Harvard  *These authors contributed equally to this work  #Corresponding Author:  Lin Tao, MD, PhD,  The First Hospital of China Medical University,  Institute of Health Sciences of China Medical University,  155 Nan Jing North Street, Shenyang 110001, Liaoning, China;  Tel/Fax: (+86) 2483283360  Email address: [taolindr@163.com](mailto:taolindr@163.com) |
| Contributions | 3b | L.T., Y.M. and J.W. conceived of the presented idea. Y.M. and J.W. developed the theory and performed the computations. K.W, W.D. and K.Y. verified the analytical methods. Z.B. and H.L. supervised the findings of this work. S.Z. made visual graphics. L.T., Y.M. and J.W. wrote the first version of the manuscript. All authors discussed the results and contributed to the final manuscript. |
| Amendments | 4 | None |
| Support: |  |  |
| Sources | 5a | None |
| Sponsor | 5b | None |
| Role of sponsor or funder | 5c | None |
| INTRODUCTION | | |
| Rationale | 6 | With the COVID-19 epidemic breakout in China, up to 25% of diagnosed cases are considered to be severe. To effectively predict the progression of COVID-19 via patients’ clinical features at an early stage, the prevalence of these clinical factors and their relationships with severe illness were assessed. |
| Objectives | 7 | Data of 3.547 patients from 24 studies was included in this study. The clinical features, and laboratory examination could be used to estimate the process of infection in COVID-19 patients. |
| METHODS | | |
| Eligibility criteria | 8 | Studies describing the epidemiological and clinical characteristics of COVID-19 cases and excluding articles of review, conference reports, editorials, letters, etc. |
| Information sources | 9 | The PubMed, Embase, Web of Science and Chinese databases. |
| Search strategy | 10 | “((COVID-19) OR (2019 nCov) OR (2019 coronavirus)) AND ((ICU) OR (severe)) AND ((clinical characteristics) OR (clinical feature))” on April 7, 2020 without limiting the language of the articles. |
| Study records: |  |  |
| Data management | 11a | Studies that included data on fewer than 10 cases were excluded. References listed in the retrieved articles were also browsed to prevent the overlooking of relevant research. |
| Selection process | 11b | According to the inclusion and exclusion criteria, two researchers independently conducted literature screening. Any disagreements between the two researchers were resolved through discussions with a third independent researcher. The retrieved articles underwent two rounds of screening, the first of which was based on the titles and abstracts, and the second of which was based on the full texts of the articles. |
| Data collection process | 11c | The data of each included study was extracted by one researcher, then verified and proofread by a second researcher. The two researchers corrected all errors through discussion and proofreading. |
| Data items | 12 | General information about the patients’ genders, clinical characteristics such as symptoms, signs, chronic comorbidities, laboratory test results and whether they were severe cases was extracted. |
| Outcomes and prioritization | 13 | The clinical features, and laboratory examination could be used to estimate the process of infection in COVID-19 patients. |
| Risk of bias in individual studies | 14 | To assess the prevalence of clinical features in COVID-19 cases, meta-analysis of single rate of each clinical feature was performed. Therefore, this meta-analysis was performed using a random effects model to prevent defferences of comorbidities between populations. |
| Data synthesis | 15a | We use the QUADAS-2 tool in Review Manager 5.2 to evaluate the quality of literature. The QUADAS-2 tool evaluates the quality of the included literature from four aspects: Patient selection, Index test, Reference standard, Flow and timing. |
|  | 15b | Cochran’s Q test was then performed with STATA 15, and the value of the degree of heterogeneity I2 was calculated to verify the correctness of the random effects model. When P ≤ 0.1 or I2 ≥ 50%, it indicated that it was correct to adopt the random effect model; otherwise, the fixed effect model was used. Finally, a forest map of comorbidities was created to show the prevalence of each clinical feature in patients. |
|  | 15c | The sensitivity, specificity, positive likelihood ratio, negative likelihood ratio and diagnostic ratio of each clinical feature, which can indicate the effect of the presence or absence of clinical features on the possibility of requiring critical care, were then calculated with a 95% confidence interval. |
| Meta-bias(es) | 16 | Publication bias analysis and sensitivity analysis were performed for each indicator to be evaluated through Stata 15. All the above calculations and drawings are realized by software Review Manager 5.3, STATA 15, Meta-DiSc 1.4 and R. |
| Confidence in cumulative evidence | 17 | To evaluate the diagnostic values of different clinical features of patients for future critical care, 2 × 2 tables were constructed for each clinical feature and each situation of being severe illness based on the extracted information. When an item in the table was empty, 0.5 was added to all cells. The sensitivity, specificity, positive likelihood ratio, negative likelihood ratio and diagnostic ratio of each clinical feature, which can indicate the effect of the presence or absence of clinical features on the possibility of requiring critical care, were then calculated with a 95% confidence interval. |
